# The Dipeptidyl Peptidase-4 Inhibitor Saxagliptin as a Candidate Treatment for Disorders of Consciousness: A Deep Learning and Retrospective Clinical Analysis

**DOI:** 10.1007/s12028-025-02217-0

**Published:** 2025-02-04

**Authors:** Daniel Toker, Jeffrey N. Chiang, Paul M. Vespa, Caroline Schnakers, Martin M. Monti

**Affiliations:** 1 Department of Neurology, University of California, Los Angeles, Los Angeles, CA USA; 2 Department of Psychology, University of California, Los Angeles, Los Angeles, CA USA; 3 Department of Computational Medicine, University of California, Los Angeles, Los Angeles, CA USA; 4 Department of Neurosurgery, University of California, Los Angeles, Los Angeles, CA USA; 5 Research Institute, Casa Colina Hospital and Centers for Healthcare, Pomona, CA USA

**Keywords:** Artificial intelligence, Disorders of consciousness, Coma, Persistent vegetative state, Minimally conscious state, Incretin

## Abstract

**Background:**

Despite advancements in the neuroscience of consciousness, no new medications for disorders of consciousness (DOC) have been discovered in more than a decade. Repurposing existing US Food and Drug Administration (FDA)—approved drugs for DOC is crucial for improving clinical management and patient outcomes.

**Methods:**

To identify potential new treatments among existing FDA-approved drugs, we used a deep learning–based drug screening model to predict the efficacy of drugs as awakening agents based on their three-dimensional molecular structure. A retrospective cohort study from March 2012 to October 2024 tested the model’s predictions, focusing on changes in Glasgow Coma Scale (GCS) scores in 4047 patients in a coma from traumatic, vascular, or anoxic brain injury.

**Results:**

Our deep learning drug screens identified saxagliptin, a dipeptidyl peptidase-4 inhibitor, as a promising awakening drug for both acute and prolonged DOC. The retrospective clinical analysis showed that saxagliptin was associated with the highest recovery rate from acute coma among diabetes medications. After matching patients by age, sex, initial GCS score, coma etiology, and glycemic status, brain-injured patients with diabetes on incretin-based therapies, including dipeptidyl peptidase-4 inhibitors and glucagon-like peptide-1 analogues, recovered from coma at significantly higher rates compared to both brain-injured patients with diabetes on non-incretin-based diabetes medications (95% confidence interval of 1.8–14.1% higher recovery rate, *P* = 0.0331) and brain-injured patients without diabetes (95% confidence interval of 2–21% higher recovery rate, *P* = 0.0272). Post matching, brain-injured patients with diabetes on incretin-based therapies also recovered at a significantly higher rate than patients treated with amantadine (95% confidence interval for the difference 2.4–25.1.0%, *P* = 0.0364). A review of preclinical studies identified several pathways through which saxagliptin and other incretin-based medications may aid awakening from both acute and chronic DOC: restoring monoaminergic and GABAergic neurotransmission, reducing brain inflammation and oxidative damage, clearing hyperphosphorylated tau and amyloid-β, normalizing thalamocortical glucose metabolism, increasing neural plasticity, and mitigating excitotoxic brain damage.

**Conclusions:**

Our findings suggest incretin-based medications in general, and saxagliptin in particular, as potential novel therapeutic agents for DOC. Further prospective clinical trials are needed to confirm their efficacy and safety in DOC.

**Supplementary Information:**

The online version contains supplementary material available at 10.1007/s12028-025-02217-0.

## Introduction

The profound challenge of restoring awareness in both acute coma patients and in patients who evolve from coma into prolonged disorders of consciousness (DOC), such as unresponsive wakefulness syndrome/vegetative state or minimally conscious states, highlights a critical gap in medical science [1, 2].

Recent efforts to develop new treatments for DOC [3] have largely been based on the mesocircuit model [4], which suggests that DOCs are “disconnection syndromes” [5] characterized by interruptions that depress excitatory long-range thalamocortical projections, leading to a broad decrease in synaptic activity and thalamocortical glucose metabolism in both acute and chronic cases [4, 6, 7]. Pharmacological strategies have aimed at restoring thalamocortical activity in patients with DOC, either through dopaminergic stimulation of striatal cells [8] or by decreasing inhibitory pallidal output [4] and countering pathological cortical hyperexcitability [9–11] via GABAergic agents. Tricyclic antidepressants have also been trialed in this cohort, owing to likely additional dysfunction in serotonergic and noradrenergic transmission in DOC [12].

The search for effective treatments for DOC is complicated by the broader sequelae of brain injuries and their temporal evolution from acute to chronic pathophysiologies. Brain inflammation, for example, occurs immediately upon severe brain injury [13–15] and can persist for years [16–18]. Additionally, both traumatic [9] and nontraumatic [10] brain injuries trigger the excessive release of glutamate, leading to both acute and long-lasting excitotoxic brain damage. Both acute [19] and chronic [20] traumatic brain injuries, as well as acute nontraumatic [21] brain injuries, are also associated with deficits in wake-promoting orexinergic neurotransmission, which affects arousal. Finally, in the long-term, severe brain injuries are linked to lower levels of brain-derived neurotrophic factor (BDNF) [22–24], which impairs neuroplasticity, as well as prolonged neurodegeneration through the accumulation of amyloid-β and hyperphosphorylated tau [25, 26], especially in patients with poor outcomes.

Collectively, the summarized findings underscore the importance of finding interventions for DOC that promote neuroplasticity, increase thalamocortical glucose metabolism, attenuate neuroinflammation, clear amyloid-β and phosphorylated tau, decrease inhibitory pallidal output, facilitate thalamocortical activity, and potentiate neurotransmission in the GABAergic, dopaminergic, noradrenergic, serotonergic, and orexinergic systems. Notably, addressing any one of these pathophysiologies is likely to impact others: decreasing pallidal output is likely to increase thalamocortical activity and glucose metabolism [4], and clearing pathological deposits such as amyloid-β is likely to facilitate neurotransmission [27] and neuroplasticity [28]. However, interventions that can directly or indirectly address all of these interconnected mechanisms are likely to be more effective for DOC than ones that target only a subset of them. Although the need for such interventions is clear, the limited availability of suitable animal models of DOC [3] (the only exception being a porcine model, which was described only recently [29]) has hindered systematic studies into whether interventions meeting these criteria can successfully treat either acute or chronic DOC. Consequently, no new candidate drugs for these conditions have been identified in more than a decade, and all marginally successful treatments to date have been serendipitously discovered and subsequently repurposed for DOC rather than being initially developed for DOC. This situation highlights a critical gap in translating our scientific understanding into practical therapeutic interventions for patients with both acute and prolonged DOC.

Because of the lack of widely available or validated animal models, drug repurposing, rather than drug development, is currently the most promising path to treatments for DOC. But this path need not rely solely on serendipity: the search for medications for DOC can be dramatically accelerated through the use of artificial intelligence. Drugs designed de novo by artificial intelligence are already being tested in human trials after demonstrating safety and efficacy in validated animal models [30]. But, even in conditions such as DOC with limited animal models, artificial intelligence can be used for drug repurposing. The power of artificial intelligence for drug repurposing was recently demonstrated in a landmark study that used a deep neural network to successfully identify a drug previously developed for diabetes as a broad-spectrum antibiotic [31]. This study used a phenotypic (or target-agnostic [32]) screen, which predicts drugs’ general efficacy based on their molecular structure rather than predicting drugs’ interactions with discrete molecular targets of interest. Such an approach may be particularly powerful in a set of conditions such as DOC, for which no clear molecular targets have been identified, and dysfunction likely spans multiple scales in the brain—from molecular abnormalities to large-scale circuit dysfunctions [4, 33]. Therefore, screening for drugs based on their demonstrated effects on the DOC phenotype rather than on predefined target interactions may help identify treatments capable of modulating multiple critical pathways simultaneously. Moreover, such phenotypic drug screens do not require in vitro evaluations of large chemical libraries; another recent study successfully identified novel senolytic drugs solely by drawing on a relatively small sample of previously published results to train a machine learning algorithm to classify molecules as either effective or ineffective senolytics [34].

**Fig. 1:**
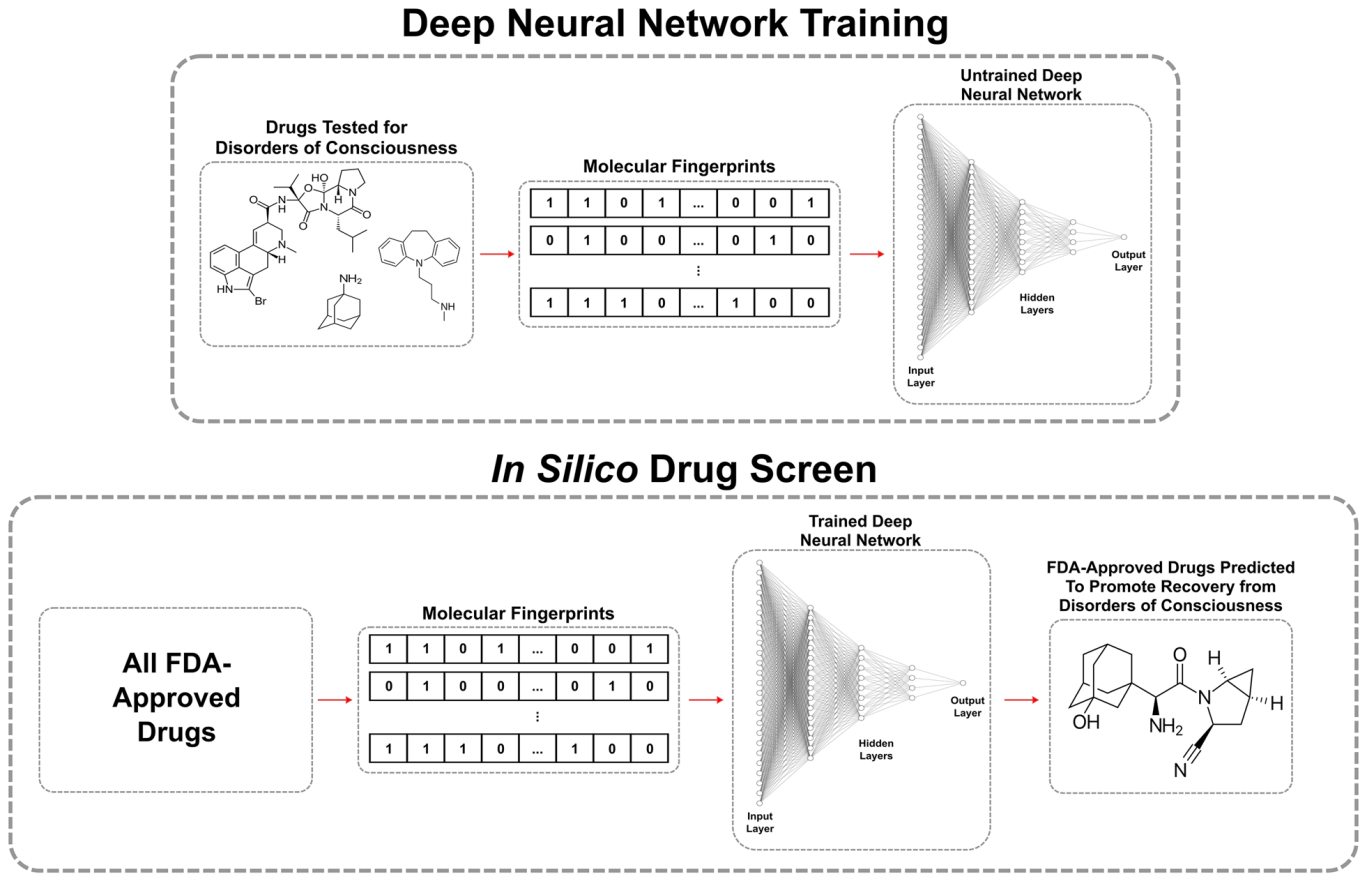
Schematic of our deep learning approach for finding US Food and Drug Administration (FDA)–approved medications with promise in treating both acute and prolonged disorders of consciousness

Here, for the first time, we employed a deep learning phenotypic drug screen [35] (Fig. 1) to identify new candidate medications for repurposing in both acute and prolonged DOC. We then conducted a retrospective cohort analysis to assess the relationship between candidate treatments and recovery outcomes in patients with acute DOC. Finally, we reviewed preclinical data to explore potential mechanisms by which these candidate drugs could influence recovery in both acute and prolonged DOC.

## Methods

### Data Collection for Deep Learning Drug Screens

To identify potential therapeutic drugs for both acute and prolonged DOC, we conducted two phenotypic drug screens using deep neural networks: one screen for prolonged DOC and one screen for acute DOC.

To compile training data for our prolonged DOC phenotypic drug screen, we conducted a comprehensive review of existing literature, including case reports, series, and placebo-controlled randomized trials that quantified clinical improvement in patients with prolonged DOC (minimum 0.9 months post injury) in response to ten medications. Those medications were zolpidem, baclofen, levodopa, bromocriptine, apomorphine, methylphenidate, modafinil, selegiline, amantadine, and desipramine, all of which have shown moderate, sporadic success in restoring awareness in prolonged DOC [12, 36] (Supplementary Table 1). Of these, only amantadine and zolpidem have been studied in randomized placebo-controlled trials, and only amantadine has been validated with a class II evidence study [37]. For this reason, steps were taken to account for the limitations of the remaining drugs in our model, as described in the Deep learning phenotypic drug screens section. We additionally compiled a separate list of 280 drugs and supplements that are likely ineffective in restoring awareness in prolonged DOC (Supplementary Table 2) as negative training data for the deep neural network. Thus, the deep neural network would be trained to accurately predict both which drugs are effective and which drugs are likely ineffective in restoring awareness in prolonged DOC.

For the second drug screen, we focused on acute DOC, and reviewed drugs that have been administered to coma patients in the intensive care unit (ICU) and have been shown to improve outcomes from both traumatic and nontraumatic brain injuries. The drugs considered were nimodipine, anatibant, progesterone, amantadine, modafinil, methylphenidate, and memantine (Supplementary Table 3). These drugs were selected based on their efficacy as measured by clinical scales. To be included, drugs needed to show efficacy in both traumatic and nontraumatic brain injury at least in animal models, with human efficacy data available for traumatic brain injury and/or nontraumatic brain injury. For all drugs but anatibant and progesterone, human efficacy data were available for both traumatic and nontraumatic brain injuries. Additionally, we included 55 failed or ineffective drugs in the acute drug screen as part of the training data (Supplementary Table 4).

For each study identified in both screens, we extracted raw changes in relevant continuous, quantitative behavioral scales of consciousness (e.g., the Glasgow Coma Scale [GCS] [38] or Coma Recovery Scale-Revised [CRS-R] [39]) for individual patients or average changes reported across study cohorts. These raw changes were contextualized within the confines of the minimum and maximum possible values for the given scale, enabling the computation of a normalized score change. This normalization process entailed calculating the proportion of maximal possible improvement achieved for each score change, thus deriving an efficacy score ranging from 0 (ineffective) to 1 (always fully effective). In instances in which the literature provided only the percentage of responders, this metric was directly adopted as an efficacy score, justified when it could be interpreted as a rough approximation of the average improvement across all study participants. The final efficacy estimate for each drug was determined by calculating the mean of all derived efficacy scores from the relevant studies. This mean efficacy score integrated data points from a multitude of clinical assessments, ensuring a representative measure of each drug’s potential therapeutic impact.

### Deep Learning Phenotypic Drug Screens

For our deep learning drug screen, we represented each drug using Simplified Molecular Input Line Entry System (SMILES) notations. The SMILES notations were then converted into Morgan molecular fingerprints [40] or extended-connectivity fingerprints [41]. The Morgan fingerprint algorithm generates binary representations of molecules by iteratively exploring the circular neighborhoods around each atom in a molecule, encoding local chemical environments by considering atom types, bond types, and hybridization states. Although it primarily captures topological structure, it also reflects electronic features indirectly through atomic properties such as formal charge, atomic number, and bonding patterns. This approach enables the identification of chemical similarities, functional groups, and substructures that can predict chemical properties and activity, making Morgan fingerprints among the best performing molecular fingerprints for in silico drug screens [42–44]. To standardize comparisons across different molecules, we forced the size of every fingerprint to be 2,048 bits, ensuring uniformity in how molecular features are compared across diverse chemical structures. Each bit in the fingerprint represents the presence (1) or absence (0) of a particular substructure or molecular feature, with different circular atom environments being encoded at various radii. This binary vector encapsulates key molecular attributes in a way that is easily processed by machine learning algorithms.

Two deep neural network models were then constructed using TensorFlow [45]. Each network was composed of an input layer shaped according to the Morgan fingerprint feature vectors, followed by three dense hidden layers with rectified linear unit (ReLU) activation functions (with 128 nodes, 64 nodes, and 32 nodes) and a final single-node dense layer for output (see Fig. 1). ReLU activation functions were chosen because of their ability to introduce nonlinearity while avoiding the vanishing gradient problem common in deep networks, thus enabling more effective learning from the input features.

The models were trained to predict a drug’s efficacy in restoring consciousness in patients with acute or prolonged DOC based on its molecular fingerprint, using a quantile loss function to address the potential overestimation of drug efficacy. This loss function was selected to make the model’s predictions more conservative, particularly focusing on the lower quantiles (0.25) of the efficacy distribution. To further mitigate bias in efficacy estimates, the estimated efficacy values of documented drugs that have been sporadically successful in treating DOC but have not been evaluated in randomized placebo-controlled trials were randomly reduced by 20% to 80% in each iteration of model training on the assumption that their reported efficacy in restoring awareness in DOC is likely strongly overestimated. The selected range introduces enough variability to reflect the uncertainty of these studies while ensuring that the potential efficacy of a drug is not excessively diminished. Moreover, the estimated efficacies of the two drugs that have shown some success for prolonged DOC in placebo-controlled clinical trials (zolpidem and amantadine) were randomly jittered in each run by 10% on the assumption that their efficacies may be slightly overestimated or underestimated. This range was chosen to balance the need for exploring variability in the estimates while minimizing the introduction of unnecessary noise. For the acute drug screen, we jittered the efficacy estimates of all drugs by ± 10%, as all drugs were evaluated in placebo-controlled studies. This approach aimed to explore a wide range of possible true efficacy values and thereby reduce the potential impact of inaccurately estimated efficacies. The models underwent 20 training iterations, each with a unique set of efficacy adjustments and a unique 80%/20% training/validation split. Across runs, the average root mean squared error (RMSE) for predicting the jittered efficacy estimates for prolonged DOC was 0.003 for the training data and 0.018 for the held-out validation data; for acute DOC, the mean training RMSE across runs was 0.0016 and the mean validation RMSE was 0.0051. For each run of the deep neural network training, the models predicted the efficacy of a comprehensive list of US Food and Drug Administration (FDA)–approved drugs (represented by SMILES notations), which were then ranked based on their predicted efficacy and molecular dissimilarity to drugs that have previously been tested on patients with DOC (estimated using the Dice similarity coefficient between the Morgan fingerprints of the tested and untested drugs).

This iterative and exploratory approach enabled the identification of chemically novel drugs that consistently demonstrated potential across various efficacy estimation scenarios in our training data, culminating in a list of top drug candidates. These candidates were distinguished by having the highest mean predicted efficacy scores, normalized by their chemical similarity to previously tested drugs for DOC, across all iterations.

### Retrospective Clinical Data in Acute DOC

Though some of the medications identified by our in silico drug screens may have potential utility in both acute DOC (i.e., coma) and prolonged DOC (minimally conscious and vegetative states), we were only able to evaluate their effects on acute coma recovery using ICU data from the University of California, Los Angeles (UCLA) medical system, which does not include patients with prolonged DOC. As a result, although we could not assess their impact on patients already in a prolonged DOC, we were able to investigate whether these medications helped prevent progression from acute to chronic DOC.

To do so, we retrospectively analyzed recovery data from patients with severe brain injuries using the Discovery Data Repository at UCLA Health. We tracked patients from their initial critical GCS score (between 5 and 8, sustained over at least 48 h) and calculated recovery rates, defined as a GCS score of 12 or higher as their final recorded GCS score or sustained over at least 3 days. We included only patients diagnosed with one of three forms of severe brain injury—namely, traumatic brain injury (*International Classification of Diseases* [ICD] code S06), vascular brain injury (ICD codes I60, I61, I62, I63, or I64), or anoxic brain injury (ICD codes G93.1, I46, or P91.6)—within 1 week before or after their recorded state of unresponsiveness to ensure that the cohort reflects DOC caused by intrinsic brain dysfunction. The initial GCS criteria of 5 to 8 sustained over at least 2 days were used to exclude patients whose low GCS scores may have been caused by transient factors, such as intubation, sedation, or other temporary clinical conditions, rather than a DOC. Recovery was defined as a GCS score of 12 or higher, either sustained over at least 3 days or recorded as the final GCS score, as most or all patients with a GCS score of 12 or higher exhibit some level of consciousness, even in the absence of full neurological recovery [46]. See the Discussion for an evaluation of the limitations of our inclusion and recovery criteria. Because the primary medication singled out by our deep learning drug screens is an incretin-based drug typically prescribed for diabetes (see Results), we specifically considered both patients who were nondiabetic and were not taking any diabetes medications and patients treated with a single diabetes medication to isolate the effect of individual drugs on recovery from coma due to severe brain injury. As a positive control, we also analyzed coma recovery rates among patients given amantadine, a leading DOC treatment, and who were not simultaneously prescribed any of the diabetes medications analyzed here. This retrospective analysis was conducted separately from the retrospective data set used during the in silico drug screen, in which we primarily examined ineffective drugs for acute DOC without consideration of comorbidities (Supplementary Table 4).

The medical charts of 1561 patients with diabetes and 2486 patients without diabetes (total = 4047) in an acute coma resulting from severe brain injury were thus retrospectively analyzed. Patient care and evaluations were conducted in the ICUs at UCLA Health. All patients were admitted to the ICUs between March 2012 and October 2024. Among the 4047 patients with acute DOC were 1720 women and 2327 men. The age at admission ranged from 2 to 100 years (mean age 65.23 years, standard deviation 19.92 years). The etiologies of coma in these patients were as follows: traumatic brain injury in 1,073 patients, vascular brain injury (nontraumatic subarachnoid hemorrhage, nontraumatic intracerebral hemorrhage, cerebral infarction, or unspecified stroke) in 2301 patients, and anoxic brain injury (cardiac arrest, hypoxic-ischemic encephalopathy, or unclassified anoxic brain damage) in 673 patients. Data extracted from patient charts included GCS scores, age, sex, etiology of coma, medication usage, and hemoglobin A1c (HbA1c) level. See Table 1 for a breakdown of the demographics of patients.

**Table 1:** Characteristics of patients in a coma from severe brain injury who are treated with various diabetes drugs or who are nondiabetic or are treated with amantadine

Drug	Patients with TBI (% recovered)	Vascular patients (% recovered)	Anoxic patients (% recovered)	Male (% recovered)	Female (% recovered)	Mean age, y	SD age, y
Acarbose	0 (N/A)	3 (66.67)	0 (N/A)	0 (N/A)	3 (66.67)	66.67	17.56
Alogliptin	0 (N/A)	3 (100)	0 (N/A)	1 (100)	2 (100)	57.33	10.69
Canagliflozin	3 (66.67)	13 (69.23)	4 (75)	13 (76.92)	7 (57.14)	66.90	14.95
Colesevelam	3 (66.67)	2 (100)	0 (N/A)	1 (0)	4 (100)	55.00	25.56
Dapagliflozin	16 (75)	51 (84.31)	31 (87.10)	72 (83.33)	26 (84.62)	63.56	16.09
Dulaglutide	4 (50)	14 (85.71)	3 (100)	15 (73.33)	6 (100)	64.86	12.77
Empagliflozin	15 (60)	46 (82.61)	15 (93.33)	53 (79.25)	23 (82.61)	62.00	15.17
Ertugliflozin	0 (N/A)	3 (100)	0 (N/A)	2 (100)	1 (100)	48.00	8.89
Exenatide	2 (100)	6 (66.67)	1 (0)	5 (80)	4 (50)	57.33	8.28
Glimepiride	9 (66.67)	43 (62.79)	12 (50)	35 (54.29)	29 (68.97)	70.44	10.57
Glipizide	36 (69.44)	104 (59.62)	33 (54.55)	105 (61.90)	68 (58.82)	69.36	12.09
Glyburide	2 (50)	23 (73.91)	4 (50)	14 (85.71)	15 (53.33)	69.93	13.65
Linagliptin	5 (80)	26 (69.23)	6 (100)	19 (73.68)	18 (77.78)	70.65	11.66
Liraglutide	2 (100)	14 (78.57)	2 (100)	11 (90.91)	7 (71.43)	61.67	12.92
Metformin	124 (65.32)	418 (65.07)	100 (59)	350 (67.71)	292 (59.93)	67.49	13.71
Nateglinide	5 (60)	16 (56.25)	5 (60)	14 (50)	12 (66.67)	72.69	10.95
Pioglitazone	10 (50)	34 (73.53)	8 (50)	29 (62.07)	23 (69.57)	69.81	11.42
Repaglinide	13 (69.23)	44 (72.73)	16 (75)	37 (72.97)	36 (72.22)	69.14	12.38
Saxagliptin	1 (100)	7 (85.71)	1 (100)	6 (100)	3 (66.67)	71.33	17.38
Semaglutide	10 (80)	26 (73.08)	3 (100)	26 (73.08)	13 (84.62)	58.18	16.34
Sitagliptin	28 (71.43)	109 (68.81)	24 (54.17)	86 (69.77)	75 (64)	69.12	14.04
All DPP-4 inhibitors	34 (73.53)	145 (70.34)	31 (64.52)	112 (72.32)	98 (67.35)	69.32	13.77
All GLP-1 analogues	30 (80)	97 (79.38)	37 (86.49)	114 (81.58)	50 (80)	61.73	15.60
Other diabetes drugs	224 (64.73)	763 (66.84)	200 (62)	668 (67.37)	519 (63.39)	67.79	13.42
Nondiabetic patients	785 (63.18)	1296 (54.17)	405 (43.70)	1433 (59.46)	1053 (49.67)	63.89	22.83
Amantadine patients	60 (51.67)	44 (68.18)	6 (16.67)	71 (54.93)	39 (58.97)	54.15	23.8

Data include the number of patients and recovery percentages stratified by TBI, vascular, and anoxic coma etiologies, as well as sex. Age is reported as mean and SD

*DPP-4* dipeptidyl peptidase 4, *GLP-1* glucagon-like peptide 1, *N/A* xxx, *SD* standard deviation, *TBI* traumatic brain injury

### Patient Matching Analysis

As part of our retrospective cohort analysis, we employed a custom multivariate patient matching algorithm. This algorithm aimed to create balanced triplets, each consisting of one patient with diabetes on an incretin-based medication, one patient with diabetes on a non-incretin-based medication, and one coma patient without diabetes. The matching process involved minimizing the total Mahalanobis distance between patients within each triplet. The Mahalanobis distance, a multivariate distance metric that accounts for correlations between variables, was calculated using age, sex, initial GCS score, coma etiology, and HbA1c as input variables. To prioritize the importance of glycemic control and age, the algorithm assigned higher weights to the HbA1c and age components of the Mahalanobis distance. Additionally, two constraints were imposed to ensure that the matched patients were sufficiently similar: first, patients in a triplet were required to have HbA1c values within the same predefined bin of 0.5%; second, the maximum allowable age difference between any two patients in a triplet was limited to a specific threshold of 5 years. By iteratively selecting the best-matching triplets that satisfied these criteria, the algorithm generated consistent and balanced patient populations across the three groups, enabling a more reliable comparison of recovery rates. For our positive control analysis, we also separately matched coma patients on incretin-based drugs to coma patients who were not on any diabetes medications and were administered amantadine. Given the large differences in baseline characteristics between incretin-treated and amantadine-treated coma patients (Table 1), patients were first matched based on coma etiology and then based on sex, age, and initial GCS score, with higher weights assigned to the sex and GCS score components of the Mahalanobis distance and the maximal allowable age difference between paired patients again set at 5 years.

## Results

### Drugs Predicted for Acute and Prolonged DOC

The deep neural network that was trained to predict the efficacy of medications that are both effective (Supplementary Table 1) and ineffective (Supplementary Table 2) in restoring awareness in prolonged DOC was used to then predict the efficacy of all FDA-approved drugs for prolonged DOC. The neural network’s top ten predictions for drugs that can be repurposed for prolonged DOC, in descending order of predicted efficacy and structural uniqueness (relative to drugs already trialed for prolonged DOC), were saxagliptin, ergotamine tartrate, imipramine pamoate, tubocurarine chloride, masoprocol, melphalan, cilastatin sodium, methylergonovine maleate, and methysergide maleate. Similarly, the second deep neural network, which was trained to predict the efficacy of both effective (Supplementary Table 3) and ineffective (Supplementary Table 4) medications for acute coma, was used to predict the efficacy of all other FDA-approved drugs for restoring brain function following coma. The top ten medications selected by this second deep learning drug screen, in descending order of predicted efficacy and structural uniqueness, were saxagliptin, brexanolone, saxagliptin hydrochloride, ganaxolone, mefloquine hydrochloride, rimantadine hydrochloride, medrysone, dydrogesterone, isradipine, and desoxycorticosterone pivalate.

Given that saxagliptin was the top prediction for both acute and prolonged DOC, we conducted a retrospective cohort analysis to evaluate whether saxagliptin and other incretin-based therapies were associated with improved outcomes following acute coma relative to other diabetes medications. See the Discussion for a review of the potential efficacy of other drugs selected by both our acute and chronic DOC drug screens.

### Retrospective Clinical Results

**Fig. 2:**
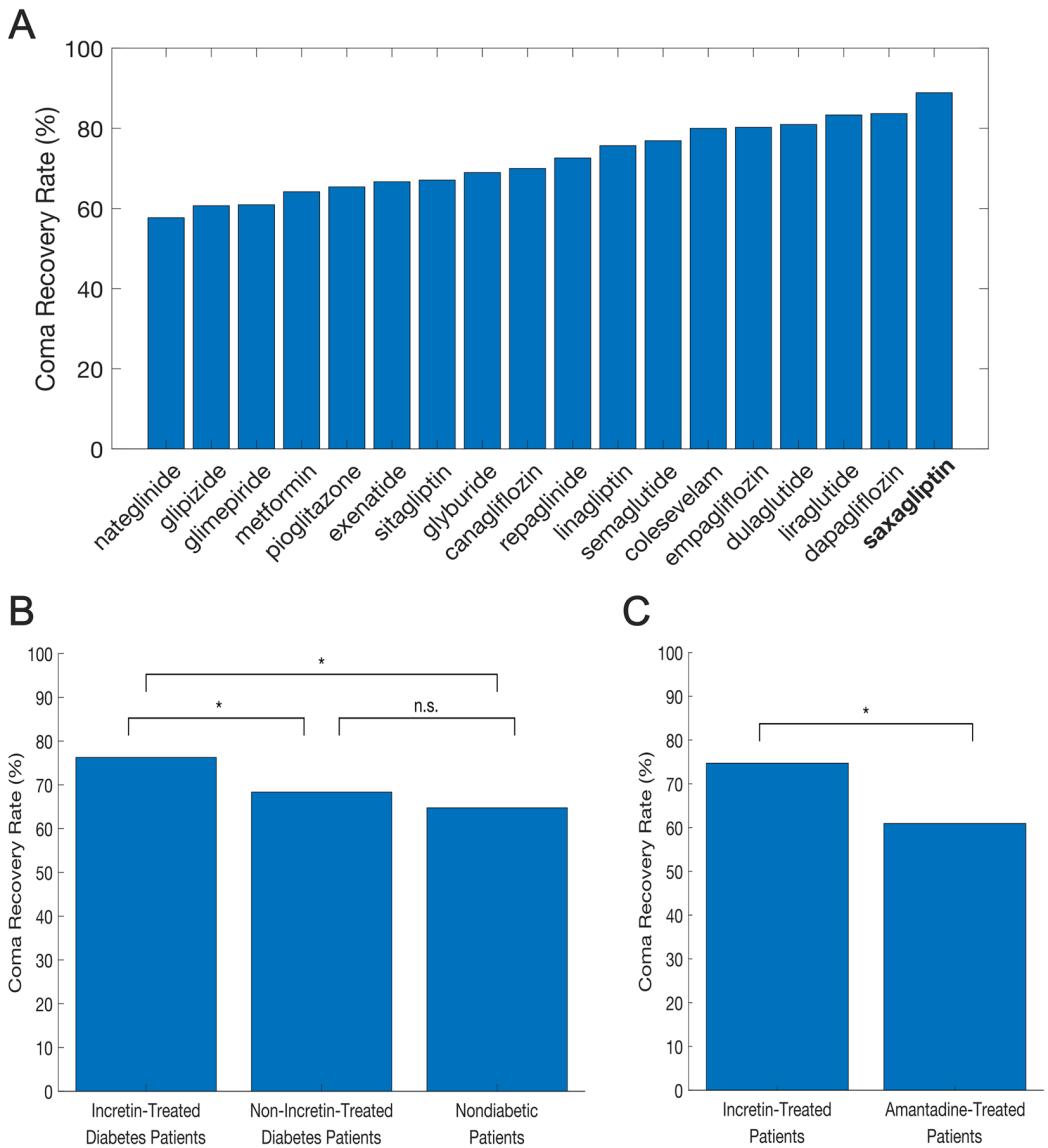
Coma recovery rates associated with diabetes medications and comparisons across patient groups. **a**, Recovery rates for coma patients (resulting from traumatic, vascular, or anoxic brain injury) treated with various diabetes medications ranked by effectiveness. Saxagliptin was associated with the highest recovery rate, although this finding is based on a small cohort of nine patients. **b**, Post matching comparisons of coma recovery rates across incretin-treated patients with diabetes, non-incretin-treated patients with diabetes, and patients without diabetes. Matching was performed based on age, sex, initial Glasgow Coma Scale (GCS) score, coma etiology (traumatic, vascular, or anoxic brain injury), and hemoglobin A1c. Incretin-treated patients showed significantly higher recovery rates compared to both non-incretin-treated patients and patients without diabetes, with no significant difference between non-incretin-treated patients and patients without diabetes (*n* = 139 patients in all three groups). **c**, Recovery rates in incretin-treated patients were significantly higher than those in amantadine-treated patients (*n* = 87 patients in both groups). For this comparison, matching was based on age, sex, initial GCS score, and coma etiology. Statistical significance was evaluated using McNemar’s paired test

Our retrospective cohort analysis corroborated the predictions of the deep neural networks: saxagliptin was associated with the highest coma recovery rate among the diabetes medications examined (Fig. 2a), though this finding is based on a small sample of just nine patients (Table 1). Of the coma patients receiving saxagliptin, two patients received 2.5 mg and all other patients received 5 mg of saxagliptin daily. Notably, all patients receiving 5 mg regained consciousness, yielding an interesting though anecdotal observation given that the sample size of coma patients taking saxagliptin was too small to infer a dose–response.

We further conducted a patient matching analysis to assess the impact of incretin-based therapies (encompassing glucagon-like peptide-1 [GLP-1] analogues and dipeptidyl peptidase-4 [DPP-4] inhibitors) in general on the likelihood of recovery from coma. In this analysis, we sought to control for potential confounders by including age, sex, initial GCS scores, coma etiology (traumatic brain injury vs. vascular brain injury vs. anoxic brain injury), and glycemic status (measured as the most recent HbA1c level taken prior to or during the coma) in a custom matching algorithm, which minimizes the distance between matched triplets of patients (one on an incretin-based drug, one on a non-incretin-based diabetes drug, and one patient without diabetes), enhancing the balance across groups and improving the comparability of the outcome (recovery from coma) (see Methods for details on the matching algorithm). This resulted in 139 patients in each of the three groups, matched by baseline characteristics and coma etiology. See Table 2 for a comparison of patient baseline characteristics and coma etiologies across the incretin, non-incretin, and nondiabetic groups.

**Table 2:** Characteristics of matched patient groups (incretin-treated, non-incretin-treated, and nondiabetic patients)

Variable	Incretin-treated patients	Non-incretin-treated patients	Nondiabetic patients	*P* value
Initial GCS score	6.70	6.71	6.81	0.3178
Age, y	66.91	67.53	67.24	0.9205
HbA1c	5.94	5.94	5.82	0.0552
Male (%)	66	65	64	0.9290
TBI (%)	18.0	14.4	16.5	0.7989
Vascular (%)	60.4	67.6	62.6	0.7989
Anoxic (%)	21.6	18.0	20.9	0.7989

This table reports the mean initial GCS score, age, and HbA1c for each group, as well as the percentage of patients in each group who are male, have a TBI, a vascular brain injury, or an anoxic brain injury. *P* values represent statistical comparisons across the 139 patients in each group, calculated using the Kruskal–Wallis test for continuous variables (initial GCS score, age, and HbA1c) and the *χ*^2^ test for categorical variables (sex and coma etiology). These results demonstrate the success of our multivariate patient matching algorithm, which minimized the Mahalanobis distance across groups based on age, sex, initial GCS score, coma etiology, and HbA1c, with additional constraints for glycemic control and age differences. The resulting balance supports the validity of pairwise comparisons of coma recovery rates across incretin-treated, non-incretin-treated, and nondiabetic patients

*GCS* Glasgow Coma Scale, *HbA1c* hemoglobin A1c, *TBI* traumatic brain injury

Post matching, the coma recovery rate for patients on incretin-based medications was 76.26%, whereas the recovery rate for patients on other antidiabetic drugs was 68.35% (95% confidence interval [CI] for the difference in recovery rates 1.8–14.1%, *P* = 0.0331, McNemar’s paired test) (Fig. 2b). Additionally, when comparing incretin-treated patients to patients without diabetes, we found a significantly higher coma recovery rate in the incretin group post matching (76.26% vs. 64.75%, 95% CI for the difference 2.0–21.0%, *P* = 0.0272) (Fig. 2b). Post hoc power analysis at an *α* of 0.1 yielded powers of 80% and 76% for the incretin vs. non-incretin and incretin vs. nondiabetic comparisons, respectively, indicating robust statistical power. Importantly, post matching, no significant difference was found in coma recovery rates between patients without diabetes and patients with diabetes receiving non-incretin-based therapies (95% CI for the difference − 5.8% to 13.0%, *P* = 0.1647) (Fig. 2b). We also conducted a second pairwise patient matching analysis to pair coma patients on incretin-based drugs to coma patients receiving amantadine, a leading DOC treatment. Owing to the limited number of amantadine-treated patients with HbA1c values on record, matching was performed based on age, sex, initial GCS score, and coma etiology, yielding 87 matched patients in the incretin group and the amantadine group (Table 3). Post matching, coma recovery rate was significantly higher among brain-injured patients treated with incretin-based drugs than among patients treated with amantadine (95% CI for the difference 2.4–25.1.0%, *P* = 0.0364, post hoc power = 76%) (Fig. 2c). These analyses suggest that incretin-based therapies may be associated with a higher likelihood of recovery from coma, irrespective of baseline patient characteristics and coma etiology, and supports the artificial intelligence–driven selection of saxagliptin specifically as a new treatment for DOC.

**Table 3:** Characteristics of matched patient groups (incretin-treated and amantadine-treated)

Variable	Incretin-treated patients	Amantadine-treated patients	*P* value
Initial GCS score	6.7	6.6	0.9278
Age	65.2	62.7	0.2614
Male (%)	40.2	35.6	0.5320
TBI (%)	47.1	47.1	1.0000
Vascular (%)	48.3	48.3	1.0000
Anoxic (%)	4.6	4.6	1.0000

This table reports the mean initial GCS score, age, and sex distribution, as well as the percentages of patients with traumatic, vascular, or anoxic brain injuries in both groups. *P* values represent statistical comparisons across the 87 matched patients in each group, calculated using the Wilcoxon signed-rank test for continuous variables (initial GCS score and age) and *χ*^2^ test for categorical variables (sex and coma etiology). These results demonstrate the success of our patient matching algorithm

*GCS* Glasgow Coma Scale, *TBI* traumatic brain injury

### Preclinical Evidence and Possible Mechanisms of Action for Acute and Prolonged DOC

Saxagliptin, which was identified by our artificial intelligence drug screens for both acute and prolonged DOC and supported by our retrospective analysis of acute DOC, has never been prospectively tested in this patient population. As a DPP-4 inhibitor, saxagliptin prevents the breakdown of incretins such as GLP-1 and glucose-dependent insulinotropic peptide (GIP), which are crucial for insulin secretion [47]. These incretins also cross the blood–brain barrier and bind to receptors expressed throughout the brain [48]. In recent years, incretin-based therapies, including DPP-4 inhibitors and GLP-1 analogues, have been explored as treatments for multiple neurological diseases. Given the absence of preclinical studies specifically on saxagliptin in DOC, we have drawn on the broader body of preclinical and clinical research into the effects of incretin-based therapies on brain function to infer possible mechanisms of action. This approach allows us to hypothesize that saxagliptin and other incretin-based drugs may promote recovery from both acute and chronic DOC through the following mechanisms.

### Restoring Neurotransmission

Incretin-based therapies such as DPP-4 inhibitors have demonstrated the ability to potentiate GABAergic, dopaminergic, serotonergic, and noradrenergic neurotransmission, which are crucial pathways for awakening drugs in both coma and prolonged DOC (see Introduction). For instance, both GLP-1 [49, 50] and GIP [51] analogues have been shown to repair dopaminergic neurons and enhance monoaminergic neurotransmission. In line with this, saxagliptin, which raises levels of both GLP-1 and GIP, has been shown to elevate brain dopamine levels in a rat model of Parkinson disease (PD) [52]; restore levels of both serotonin and dopamine in rodents exposed to anesthesia post stroke [53]; and normalize serotonin, dopamine, and norepinephrine levels in chronically stressed rats [54]. These findings suggest potential mechanisms through which incretin-based therapies may influence neurotransmitter systems relevant to recovery from DOC, as disruptions to monoaminergic pathways are a known contributor to impaired consciousness. Although these results are derived from animal models of neurological disorders such as PD and stroke, which do not directly replicate the pathophysiology of DOC, they highlight the potential of saxagliptin and other incretin-based medications to modulate monoaminergic systems that are also implicated in DOC. Similarly, DPP-4 inhibitors have also shown promise in managing Huntington disease [55] and epilepsy owing to their significant potentiation of GABAergic neurotransmission, likely via their indirect GLP-1 receptor agonism [56]. Although the GABAergic potentiation of incretin-based drugs has not yet been explored in the context of severe brain injury or DOC, these results from other neurological indications may translate to the context of severe brain injury. Finally, in vitro work has found that in addition to degrading GLP-1 and GIP, DPP-4 also degrades orexin B [57], and so it is reasonable to predict that a DPP-4 inhibitor like saxagliptin will also raise circulating levels of this orexin, though the relevance of this to DOC is unclear, as only orexin A deficiency has been studied in the context of acute and chronic brain injury [19–21]. Collectively, these findings highlight one key mechanism by which DPP-4 inhibitors like saxagliptin may promote recovery from DOC, namely the potentiation of neurotransmitter systems thought to be disrupted in these disorders, though further research is needed to confirm these effects specifically in the context of impaired consciousness.

### Promoting Neural Repair and Plasticity

Incretin-based therapies like saxagliptin have shown potential in enhancing neuroplasticity, neurogenesis, and the proliferation of neural progenitor cells, which are essential for recovery from neurological impairments. A negative correlation has been shown between DPP-4 activity and BDNF levels in elderly individuals, indicating that higher DPP-4 activity could lead to lower BDNF and increased cognitive decline [58]. This finding suggests that saxagliptin’s ability to modulate DPP-4 activity might generally enhance BDNF levels. Indeed, saxagliptin has been shown to increase BDNF levels in the specific indications of PD and Alzheimer disease (AD) [52, 54]. Similarly, vildagliptin, another DPP-4 inhibitor, also promotes BDNF expression, supporting the neuroprotective role of this drug class [55, 59]. Though the effect of DPP-4 inhibitors on BDNF has not been specifically studied in the context of DOC or severe brain injury, it is reasonable to infer that similar mechanisms could be beneficial in promoting recovery of consciousness given the known role of BDNF in neuroplasticity and neural repair across various neurological conditions. Moreover, GLP-1 receptor agonists in general enhance long-term potentiation, which is a critical aspect of neural plasticity [60, 61], though this too remains to be studied specifically in the context of DOC. Incretin-based therapies also promote axonal regeneration and functional neural repair after nerve injury [62, 63] and trigger neurogenesis and the proliferation of neuronal progenitors in the subventricular zone [64], processes that may be critical in recovery of consciousness from severe brain injuries.

### Reducing Brain Inflammation and Oxidative Damage

Incretin-based treatments, including GLP-1 and GIP analogues as well as DPP-4 inhibitors like saxagliptin, have been shown to reduce brain inflammation and oxidative stress in various neurological disorders. GLP-1 mimetics, such as exenatide and liraglutide, have demonstrated neuroprotective effects by mitigating brain inflammation in AD [65]. Incretin-based agents work by counteracting proinflammatory cytokines such as interleukin-1β, which are linked to decreased neuronal transmission and increased apoptosis [66]. Additionally, GIP receptor agonists, including D-Ala2GIP, show promise in reducing chronic neural inflammation, oxidative stress, and neural DNA damage in AD models [67, 68]. Similarly, in PD models, GIP analogues have also been shown to significantly reduce brain inflammation [69]. DPP-4 inhibitors, by elevating both GLP-1 and GIP levels, have likewise demonstrated efficacy in alleviating oxidative stress and neuroinflammation in AD and traumatic brain injury models [70–72]. Saxagliptin specifically has also been shown to promote cell viability by reducing inflammation and oxidative stress in a stroke model [73]. Though several of these findings come from conditions other than severe brain injury, they do point to a possible mechanism by which saxagliptin and other incretin-based treatments could have therapeutic value in DOC.

### Restoring Thalamocortical Glucose Metabolism

Recent research has explored the impact of incretin-based therapies on cerebral glucose metabolism, particularly within the thalamocortical system, where glucose metabolism is reduced in patients with DOC [4, 74]. For example, research by Daniele and colleagues on prediabetic patients demonstrated that a single injection of the GLP-1 receptor agonist exenatide significantly increased glucose metabolism in the cortex compared to a placebo [75]. Similarly, Gejl and colleagues showed that GLP-1 enhances the cerebral metabolic rate for glucose, particularly in the thalamus and cortex, in healthy human males [76]. Similar enhancements in brain glucose metabolism were observed with the DPP-4 inhibitor Gramcyclin A in AD mouse models [77]. In the mesocircuit hypothesis of DOC, depressed thalamocortical glucose metabolism is partly attributed to excessive inhibitory output from the globus pallidus [4]. Although the effect of incretin-based medications on pallidal activity has not been directly studied, individuals with genetically reduced GLP-1 receptor expression have been shown to exhibit pathologically increased pallidal activity during reward-related tasks [78], suggesting that incretin-based therapies may also impact thalamocortical metabolism by modulating pallidal output. Collectively, these findings underscore the potential of incretin-based drugs like saxagliptin as awakening drugs for DOC given the relationship between cerebral metabolism and pathological unconsciousness [7].

### Clearing Amyloid and Tau Pathologies

DPP-4 inhibitors, including saxagliptin, have shown potential in reducing amyloid-β and tau pathologies, which are common in prolonged DOC and severe brain injuries [79, 80]. Prior findings indicate that saxagliptin and similar drugs can effectively clear these pathological deposits in animal models of AD [70, 71, 81]. Notably, patients with diabetes with AD-related cognitive impairment using DPP-4 inhibitors exhibit a lesser amyloid burden and slower cognitive decline compared to patients without diabetes or patients with diabetes taking other glucose-lowering medications [82]. Both preclinical [83] and clinical [84] studies have also shown that GLP-1 agonists significantly reduce these pathological deposits in the brain, with similar results for GIP and dual GIP/GLP-1 agonists [67, 68]. The superior efficacy [85] of dual agonists in reducing amyloid plaque levels highlights the promise of saxagliptin, which raises both GLP-1 and GIP levels, in clearing these neuropathologies. Though these studies have primarily focused on AD, the significant potential of incretin-based medications in modulating amyloid and tau pathologies, which are a part of the pathophysiology of severe brain injury, points to another plausible mechanism by which these medications might promote recovery from DOC.

### Preventing and Reversing Excitotoxic Neural Damage

Incretin-based therapies, including GLP-1 and GIP analogues, have proven effective in ameliorating excitotoxic neuronal damage, a common consequence of both traumatic and nontraumatic brain injuries [9, 10]. Liraglutide, a GLP-1 analogue, and twincretin, a dual GLP-1/GIP analogue, enhance cell viability in neuronal cultures exposed to glutamate excitotoxicity [86, 87]. Similarly, exenatide prevents rapid ion influx, cellular enlargement, and glutamate-induced apoptosis [88, 89]. Additionally, DPP-4 inhibitors, including saxagliptin, have been shown to activate pathways that suppress proapoptotic factors, thereby enhancing neuronal survival and resilience [52, 55, 90]. Significantly, GLP-1 analogues have not only prevented but also reversed neural damage in rodent models of excitotoxic neurodegeneration [89]. Finally, there is some evidence that DPP-4 inhibitors can increase expression of insulin-like growth factor 1 (IGF-1) in brain tissue [91], which may be critical in the context of DOC, as increased expression of IGF-1 has been suggested to ameliorate excitotoxic brain damage [92].

### GLP-1-Independent Effects

It is possible that saxagliptin and perhaps other DPP-4 inhibitors may exert GLP-1-independent therapeutic effects in DOC. The potentially GLP-1-independent neurological benefits of DPP-4 inhibitors might relate to their effects on other DPP-4 substrates, such as GIP and neuropeptide Y, both of which are neuroprotective [93], or orexin B [57]. Further, saxagliptin in particular might affect nervous tissue directly, beyond its DPP-4 inhibitory effects. Notably, saxagliptin does cross the blood–brain barrier to a limited extent in non-brain-injured animals [94] and also has the highest Central Nervous System Multiparameter Optimization Score among the FDA-approved DPP-4 inhibitors (Table 4), suggesting the highest likelihood of brain penetration [95]. We also note that severe brain injury is associated with increased blood–brain barrier permeability [96, 97], which may further increase brain penetration of saxagliptin. To the degree to which it does penetrate the brain, saxagliptin may exert neuroprotective effects independent of its inhibition of DPP-4, owing to its possession of a possibly neuroactive adamantyl moiety [98], similar to that of amantadine (a leading DOC treatment).

**Table 4:** Predicted central nervous system penetration of FDA-approved DPP-4 inhibitors

Drug	Basic, pKa	Partition coefficient (logP)	Distribution coefficient at pH 7.4 (logD)	Molecular weight (Da)	Hydrogen bond donors	Total polar surface area (Å^2^)	CNS MPO
Saxagliptin	7.90	− 0.14	− 0.20	315.19	3	90	5.1667
Linagliptin	8.6	1.99	− 0.16	472.23	2	113	3.6317
Alogliptin	9.47	1.84	− 1.35	339.17	2	94	4.6317
Sitagliptin	8.78	1.30	1.09	407.12	2	77	4.7734

CNS MPO scores [95] were calculated for each US Food and Drug Administration–approved DPP-4 inhibitor to evaluate likelihood of brain penetration. A higher score means a higher likelihood of crossing the blood–brain barrier. For these calculations, basic dissociation constant (pKa) scores were derived from PubChem [99], and all other parameters were derived from the ACD/Labs Percepta Platform—PhysChem Module on ChemSpider [100]. Based on its CNS MPO score, saxagliptin is the most likely of the DPP-4 inhibitors to cross the blood–brain barrier

*CNS MPO* Central Nervous System Multiparameter Optimization, *DPP-4* dipeptidyl peptidase 4

## Discussion

This study leveraged artificial intelligence to identify saxagliptin, a DPP-4 inhibitor, as a potential treatment for both acute and prolonged DOC and provided preliminary evidence for its potential efficacy for acute DOC in retrospective clinical data. Our findings reveal that saxagliptin and other incretin-based drugs are associated with higher recovery rates from coma in patients with diabetes compared to other diabetes medications and that coma recovery rates are significantly higher among brain-injured patients with diabetes on incretin-based drugs than among patients without diabetes or patients treated with amantadine. Our review of preclinical and clinical evidence suggests that incretin-based drugs may aid recovery from both acute and chronic DOC through their effects on neurotransmission, brain inflammation, neuroplasticity, thalamocortical glucose metabolism, the clearance of pathological deposits, and ameliorating excitotoxic brain damage, and that saxagliptin in particular may exert additional neuroprotective effects because of its possession of an adamantyl moiety and higher likelihood of crossing the blood–brain barrier compared to other DPP-4 inhibitors. In relation to the broader literature, our study builds on existing preclinical and clinical research that supports the therapeutic potential of incretin-based therapies, such as saxagliptin, in neurological conditions. Prior studies have demonstrated the neuroprotective effects of incretin-based medications across a range of neurological disorders, including PD, AD, epilepsy, stroke, and traumatic brain injury [52, 56, 68, 70–72, 101]. Although our study is the first to apply these insights to the DOC population, it aligns with the growing body of evidence suggesting that incretin-based therapies offer significant neuroprotective benefits, providing a strong rationale for their investigation in DOC. It is also notable that bromocriptine, a dopaminergic agent that also raises endogenous levels of GLP-1 [102], is one of the few medications to show promise in enhancing levels of awareness in chronic DOC [103], which further underscores the need to more carefully study the potential role of incretin-based drugs in DOC treatment.

Despite these promising results, significant limitations must be addressed. The retrospective design of our analysis of acute coma patients limits our ability to establish causality between saxagliptin/incretin-based medication use and improved DOC outcomes, and the small sample size for saxagliptin specifically makes it difficult to draw definitive conclusions about its effectiveness. Indeed, although saxagliptin was the top in silico prediction for both acute and chronic DOC, stands out among incretin-based drugs for its possession of a (possibly) neuroactive adamantyl moiety, and stands out among DPP-4 inhibitors more specifically for its higher probability of brain penetration, our retrospective analysis mainly supports the potential efficacy of incretin-based therapies broadly rather than saxagliptin specifically. Moreover, our retrospective analysis could only provide direct empirical support for the potential efficacy of incretin-based therapies for acute DOC rather than chronic DOC, though the recovery of consciousness following coma precludes the evolution from acute to chronic DOC. It is also possible that the observed effects in our retrospective clinical analysis for incretin-based drugs in general might be partially influenced by factors not controlled for in this study, such as variations in medical care practices or unmeasured patient health variables. The variability in DOC etiology and progression further complicates the application of a single-drug intervention across all cases. Moreover, repurposing any incretin-based medications, which are not currently approved for treating DOC, raises substantial ethical and safety concerns. Given the vulnerability of patients with DOC, the off-label use of saxagliptin or other incretin-based drugs must proceed with extreme caution, under the guidance of rigorous ethical standards.

Another limitation of our study relates to the selection of drugs used in our phenotypic drug screen for prolonged DOC. Although all effective medications used for our acute DOC drug screen have support from randomized placebo-controlled clinical trials, many of the medications included for the prolonged DOC screen, such as baclofen and desipramine, have shown only sporadic efficacy in case studies. To account for this, we mitigated potential bias by adjusting the efficacy estimates of these drugs during model training (see Methods and Deep Learning Phenotypic Drug Screens sections), thereby reducing the likelihood of overestimating the effects of drugs with weaker evidence. However, it remains possible that the inclusion of these medications influenced the final outcomes of the model, and this must be considered when interpreting the results. Additionally, a significant limitation arises from the fact that the studies from which we derived drug efficacy scores used a variety of behavioral assessments, each of which captures overlapping but distinct features of arousal, awareness, and cognition. Although we attempted to mitigate this issue by normalizing efficacy estimates and averaging across studies, this is a limited approach and does not fully account for the variability in outcome measures. In addition, we note that the reviewed clinical studies from which we derived our drug efficacy estimates (and on which our deep neural network was trained) included patients with DOC with both traumatic and nontraumatic brain injuries. However, this should not affect the accuracy of our deep learning approach, as treatments consistently demonstrate no notable differences in efficacy between DOC etiologies [104–110]. Moreover, although traumatic versus nontraumatic DOC etiologies differ in their initial mechanisms, they frequently result in similar patterns of thalamic atrophy [111, 112] and depressed thalamocortical glucose metabolism [6]. As such, the inclusion of medications used for patients with both traumatic and nontraumatic injuries in our drug screens is warranted given the common pathophysiological features across DOC populations.

Another limitation of our study is the nature of the data available for the retrospective analysis. First, reliance on the GCS to define coma and recovery of consciousness can be problematic because although it is easy and rapid to implement in the intensive care environment, this scale was not designed to identify subtle behaviors (e.g., visual pursuit, fixation) crucial for detecting consciousness [113, 114]. We thus cannot exclude the possibility that some patients considered to be in a state of coma on the basis of a GCS score of 8 or lower did in fact possess some undetected level of awareness. Similarly, although we considered patients with a GCS score of 12 or above to be recovered owing to the fact that patients in this GCS range typically demonstrate some level of consciousness as assessed by the CRS-R, we cannot exclude the possibility that some patients with lower scores also possessed some level of undetected awareness [46]. Finally, to avoid including patients who were in a state of coma due to transient factors (e.g., sedation), we excluded patients with a GCS score of 3 or 4. Although this heuristic likely reduces unwanted sources of variance, it also risks biasing our results by potentially omitting the very severe end of the brain injury spectrum by excluding patients who present with low GCS scores (i.e., 3, 4) in the absence of any sedation. These limitations highlight the importance of confirming our retrospective findings with a prospective investigation making use of more appropriate neurobehavioral assessments (e.g., CRS-R).

In future work, it will also be valuable to explore the medications singled out by our in silico drug screens, other than saxagliptin. For the acute DOC drug screen, these were brexanolone, ganaxolone, mefloquine hydrochloride, rimantadine hydrochloride, medrysone, dydrogesterone, isradipine, and desoxycorticosterone pivalate. Most of these drugs are neurosteroids that potentiate GABAergic neurotransmission and may therefore be beneficial in mitigating excitotoxic brain damage following acute severe brain injury: brexanolone and ganaxolone both have antiseizure effects owing to their potent allosteric modulation of γ-aminobutyric acid A (GABA-A) receptors [115]; dydrogesterone has been shown to increase brain levels of allopregnanolone [116], which shares the neuroprotective effects of progesterone relevant to recovery from severe brain injury but, unlike progesterone, also positively modulates GABA-A receptors (though progesterone also indirectly modulates GABA-A receptors through its conversion into allopregnanolone) [117]; and desoxycorticosterone pivalate is metabolized into neurosteroids, which are also potent allosteric modulators of GABA-A receptors [118, 119]. Importantly, most of these neurosteroids are all also likely to mitigate the acute and chronic inflammation that results from severe brain injury [120–122]. Two of the other drugs singled out by our screen, mefloquine and isradipine, have more direct evidence supporting their ability to mitigate excitotoxic brain damage: mefloquine, an antimalarial drug, has been shown to prevent excitotoxicity-induced neuronal death in animal models of both stroke [123] and traumatic brain injury [124], owing to its potent blockade of Cx36-containing gap junctions. Mefloquine is also an agonist of the 5-HT_2a_ receptor [125], which may help address the disruption of serotonergic transmission in acute brain injury [126]. Isradipine is an L-type calcium channel blocker (like nimodipine), which has also been shown to mitigate glutamate-induced excitotoxicity [127], which likely underpins its therapeutic promise in stroke [128, 129] (though see ref [130]); in addition, isradipine has shown some therapeutic potential in PD [131] and AD [132]. The remaining two top predicted medications—medrysone and rimantadine—have no known mechanisms by which they might mitigate excitotoxicity. However, recent evidence [133] suggests that medrysone inhibits astrogliosis and could therefore be beneficial for coma recovery from both traumatic brain injury [134] and stroke [135] while also promoting myelin repair, which could be particularly advantageous in cases of coma caused by diffuse axonal injury. Finally, rimantadine hydrochloride, which was also singled out by our drug screen, has shown some preliminary efficacy in treating PD [136, 137], but evidence for its efficacy in other neurological indications or its neuroactive mechanisms is otherwise limited.

For the chronic DOC drug screen, the top identified medications, other than saxagliptin, were ergotamine tartrate, imipramine pamoate, tubocurarine chloride, masoprocol, melphalan, cilastatin sodium, methylergonovine maleate, and methysergide maleate. Both ergotamine tartrate and methysergide maleate are ergot alkaloids and are agonists of α-adrenoceptors, dopamine D_2_ receptors, and several 5-HT_1_ receptors [138] and thus may help DOC by modulating multiple subcortical arousal systems. Methylergonovine maleate is also an ergot alkaloid, which, in addition to being an agonist at the same receptors as ergotamine and methysergide, is also an agonist at 5-HT_2_ receptors [139], which likely underpins its mild psychedelic effect [140], a feature that is notable in the context of DOC in light of recent theories suggesting a therapeutic benefit of psychedelics in these disorders [141, 142]. Imipramine pamoate, another drug predicted by our in silico screen as effective for chronic DOC, is metabolized into desipramine [143], a tricyclic antidepressant that has also shown some limited efficacy in restoring awareness in these conditions [144]. Masoprocol is a form of nordihydroguaiaretic acid, a potent antioxidant and lipoxygenase inhibitor that has shown neuroprotective effects against oxidative stress [145] and ischemia/reperfusion injury [146, 147] and thus may offer some benefit in DOC. The remaining drugs, cilastatin sodium, tubocurarine, and melphalan, are less well understood in the context of brain function. Cilastatin sodium does penetrate into cerebrospinal fluid [148] and inhibits dehydropeptidase [149], the activity of which is enhanced in the presence of astrogliosis [150], a hallmark of both TBI [134] and stroke [135], though the therapeutic benefit of inhibiting dehydropeptidase in the brain has not been studied for any indication to our knowledge. The mechanisms by which tubocurarine may promote recovery of awareness is unclear, though it is interesting that, like baclofen (which has shown some efficacy in DOC [151–154]), tubocurarine is a muscle relaxant, though it is thought to work through entirely different mechanisms [155]. Finally, melphalan, although able to cross the blood–brain barrier [156], has no known mechanism by which it might impact DOC recovery.

It is also important to highlight the safety and risks associated with saxagliptin use, particularly in light of the vulnerability of patients with DOC. Although saxagliptin has demonstrated a generally favorable safety profile in patients with type 2 diabetes [157], certain concerns merit careful consideration. Most notably, the SAVOR-TIMI 53 trial revealed an increased risk of hospitalization for heart failure in saxagliptin-treated patients, with a hazard ratio of 1.27 compared to placebo [158]. However, follow-up analyses have failed to replicate this finding [159]. Moreover, saxagliptin does not appear to increase the risk of ischemic events, pancreatitis, or serious adverse outcomes such as arthralgia compared to placebo or other antidiabetic drugs [157–159]. Furthermore, saxagliptin has been associated with a better safety profile relative to sulfonylureas, particularly regarding the risk of hypoglycemia [159]. However, the FDA has recommended that saxagliptin be discontinued in patients who develop heart failure, underscoring the need for vigilance in its use [160]. For patients with DOC, the cardiovascular vulnerability associated with their critical condition may amplify these risks, and careful patient monitoring would be essential in any clinical application of saxagliptin.

Particularly in light of the limitations reviewed, our findings suggest the need for prospective randomized controlled trials to confirm the safety and efficacy of saxagliptin and other incretin-based drugs, as well as some of the other drugs identified by our in silico screens in both acute and prolonged DOC. Future trials will be necessary to determine the optimal doses of any of these medications for DOC. For saxagliptin, the results presented herein suggest 5 mg/day as a possibly effective dosage, as all coma patients receiving this dosage recovered consciousness (though the sample size is too small to establish a dose effect). Moreover, in animal models of other neurological conditions [52, 54, 70], saxagliptin has shown neuroprotective effects at dosages translating to human equivalents (based on body surface area [161]) of 2.84–22.68 mg/day for a 70-kg human, which suggests that the 5-mg dose falls within the range expected to exert neuroprotective effects, at least in other indications. Given these promising findings in other contexts, as well as our deep learning and retrospective results, clinical trials of saxagliptin in patients with DOC are essential to determine whether similar benefits may be observed. These trials should be designed with diverse patient populations to ensure results are generalizable and should adhere to the highest standards of research ethics and participant safety. Such trials may also help elucidate whether the beneficial effects observed are due to the specific actions of saxagliptin or result from broader pharmacological properties shared among incretin-based therapies. Further investigation of saxagliptin and other incretin-based therapies, as well as some of the other medications singled out by our in silico drug screens, may potentially transform care standards for DOC and improve patient outcomes.

## Source of Support

This research did not receive any specific grant from funding agencies in the public, commercial, or not-for-profit sectors. However, the first author, Daniel Toker, is currently receiving funding from the National Institute of General Medical Sciences for an unrelated project.

## Conflicts of interest

The authors have no competing interests to declare.

## Electronic Supplementary Material

The online version of this article (https://doi.org/10.1007/s12028-025-02217-0) contains supplementary material.

## Supplementary Information

Below is the link to the electronic supplementary material.

Supplementary file1 (PDF 115 KB)

## Data Availability

The data used to train our deep neural networks are available in Supplementary Tables 1–4.
