# Supplementary material for: The Dipeptidyl Peptidase-4 Inhibitor Saxagliptin as a Candidate Treatment for Disorders of Consciousness: A Deep Learning and Retrospective Clinical Analysis
Source: Neurocrit Care. 2025 Feb 4;43(1):101–18. doi: 10.1007/s12028-025-02217-0 (PMC12321933; doi:10.1007/s12028-025-02217-0)
Supplement: Supplementary file 1 — Supplementary file1 (PDF 115 KB) [file 12028_2025_2217_MOESM1_ESM.pdf]

Supplementary Information

Supplementary Table 1. Estimates for the efficacy of ten drugs—amantadine, zolpidem, apomorphine, bromocriptine, levodopa, baclofen, desipramine, methylphenidate, modafinil, and selegiline—in improving awareness in prolonged DOC were extracted from a range of controlled trials and case reports. Because of the heterogeneity of outcome measures across studies, a normalized efficacy score for each drug was calculated as the raw score change (either for a single patient or averaged across patients) divided by the maximum possible score for a given scale minus the minimum possible score for that scale. In cases for which changes in clinical scales were not directly reported, the percentage of responders—defined as the proportion of patients achieving clinically significant improvements in awareness, as reported in the original studies—was instead adopted as the normalized score change. For each drug, a mean normalized efficacy score was calculated and used to train the deep neural network. Note that a negative normalized change value for a single patient denotes worsening of consciousness or cognitive function for that patient. CNC = Coma/Near Coma Scale, CRS-R = Coma Recovery Scale-Revised, DRS = Disability Rating Scale, GCS = Glasgow Coma Scale, Rancho = Rancho Los Amigos Scale, GOS-E = Glasgow Outcome Scale-Extended, CRS = Coma Recovery Scale, Wessex = Wessex Head Injury Matrix Score. "Custom" here indicates that a study used its own assessment tool to evaluate improvements in consciousness/cognition, rather than a standard scale.

**Drug**	**Ref**	**Outcome measure**	**Placebo-controlled**	**Min time since injury**	**Raw score change**	**Normalized change**
Amantadine	1	CNCS	no	5 months	3.41	0.853
	2	CRS-R	no	2 years	3.5	0.152
	3	DRS	yes	1 month	4.75	0.164
						**Average= 0.389**
Zolpidem	4	GCS	no	3 years	6	0.5
					4	0.333
					5	0.417
	5	Rancho	no	18 months	2	0.222
	6	CRS-R	yes	1 month	6.5	0.283
					-3.5	-0.152
					-2	-0.087
					-1.5	-0.065
					-1	-0.043
					-1	-0.043
					-1	-0.043
					-0.5	-0.022
					-0.5	-0.022
					-0.5	-0.022
					0	0
					0	0
					0	0
					0.5	0.022
					1	0.043
	7	Rancho	yes	2 years	0	0
					0	0
					0	0
	8	% Responders	yes	4 months		0.048
	9	% Responders	no	1 month		0
						**Average= 0.057**
Apomorphine	10	GOS-E	no	3.4 months	7	1
	11	CNCS	no	1.5 months	20	0.455
					33	0.75
					41	0.932
					38	0.864
					36	0.818
					26	0.591
					37	0.841

	12	CRS-R Index	no	2 months	16.84	0.168
					13.55	0.136
					25.86	0.259
					30.2	0.302
					39.78	0.398
					70.86	0.709
						**Average= 0.587**
Bromocriptine	13	CRS	no	1 month	12	0.48
						**Average= 0.48**
Levodopa	14	GCS	no	1 month	5	0.417
	15	custom	no	1 month	4	0.8
					5	1
					5	1
					1	0.2
					1.5	0.3
					5	1
					5	1
					4.5	0.9
					2	0.4
					0	0
					5	1
						**Average= 0.668**
Baclofen	16	CRS-R	no	6 months	1	0.043
					14	0.609
					14	0.609
					6	0.261
					6	0.261
	17	CRS-R	no	5 months	0	0
					0	0
					0	0
					1	0.043
					0	0
					0	0
					0	0
					9	0.391
					11	0.478
	18	CRS-R	no	14 months	6	0.261
	19	CRS-R	no	.9 months	6.4	0.278
						**Average= 0.202**
Desipramine	20	Rancho	no	19 months	3	0.333
						**Average= 0.333**
Methylphenidate	21	GCS	no	1 month	12	1
	22	GCS	no	1 month	2	0.167
					3	0.25
					2	0.167
					4	0.333
					1	0.083
					4	0.333
					3	0.25
					2	0.167
					5	0.417

					3	0.25
					3	0.25
					2	0.167
					2	0.167
					4	0.333
						**Average= 0.289**
Modafinil	23	Wessex	no	2 months	1	0.016
					1.5	0.024
					1	0.016
					-0.5	-0.008
					10	0.161
					1.5	0.024
					3	0.048
					1	0.016
					7.5	0.121
					23	0.371
					8	0.129
					3	0.048
					0.04	0.001
					5	0.081
					2	0.032
					4	0.065
					3	0.048
					5	0.081
					-1	-0.016
					0	0
					0	0
					-1	-0.016
					8.5	0.137
						**Average= 0.057**
Selegiline	24	CRS-R	no	6 months	11	0.478
					7	0.304
					7	0.304
					6	0.261
					0	0
					-2	-0.087
					0	0
					2	0.087
					1	0.043
					1	0.043
						**Average= 0.1433**

Supplementary Table 2. Drugs and supplements that are ineffective, or presumed to be ineffective, in restoring awareness in prolonged DOC. Data are based on one published report[25], as well as records from 20 patients in prolonged DOC, evaluated over multiple visits by co-author Caroline Schnakers. These patients had remained stably unconscious for several months, and the drugs and supplements listed were recorded as part of their treatment regimens during these visits. Due to the chronic nature of their condition, we infer that these drugs and supplements likely do not contribute to recovery of consciousness, though this inference is based on observational data and should be interpreted with caution.

**Drugs from Patient Records**	**Drugs from Bagnato et al.**[25]
Abilify, Accucheck, Acculate, Acetaminophen, Acetylcysteine, Acidophilus, Albalon, Albuterol, Align, Almacone, Alphafetroprotein, Amikacin, Amlodipine, Anusol, Apresoline, Arginaid, Aricept, Arquanaid, Ascorbic, Aspirin, Atenolol, Ativan, Atropine, Atrovastatin, Atrovent, Augmentin, Avandia, Bacitracin, Bactrim, Benadryl, Benefiber, Beneprotein, Benzotropine, Benzoyl, Betamethasone, Biotene mouth spray, Bisac Evac, Bisacodil, Bisacodyl, Biscolax, Botox, Calcium, Caldesene, Calmoseptine, Carafate, Cardiazem, Cardizem, Catapres, Catapress, Cefepime, Ceftazidime, Cerovite, Chloredixine, Chlorexidine, Chlorhexadine, Chlorhexidine, Chromium Picolinate, Cipro, Ciprofloxacin, Claritin, Cleacin, Clindamycin, Clonidine, Colace, Compazine, Coumadin, Cranberry, Cyanocobalamin, Cymbalta, Daliresp, Dantrium, Dantrolene, Depakote, DHA, Diazepam, Diflucan, Digox, Digoxin, Dilantin, Diltiazem, Diphenhist, Diphenhydramine, Docosahexanoic, Docusate sodium, Doxycicline, DSS, Ducosate, Dulcolax, Duoderm, Duoneb, Effexor, Elixophyllin, Enema, Enoxaparin, Ergocalciferol, Eucerin, Famotidine, Fentanyl, Finasteride, Flexeril, Floranex, Florastor, Flulaval, Fluticasone, Folic Acid, Gabapentin, Gentamicin, Glipizide, Glucerna, Glucotrol, Glycopirolate, Glycopyrrolate, Guaifenesin, Haldol, Heparin, Humulin, Hydralazine, Hydroclotorothiazide, Hydrocodon, Hydrocodone, Hydrocodone-Acetaminophen, Hydrocortisone, Hyoscyamine, Ibuprofen, Imodium, Inderal, Insulin, Ipratropium, Ivermectin, Kaopectate, Keflex, Keppra, Klonopin, Lactic Acid, Lactobacillus, Lactulose, Lansoprazole, Levalbulterol, Levalbuterol, Levaquin, Levemir, Levothyroxine, Lexapro, Lipitor, Lisinopril, Loperamide, Lopressor, Loratadine, Lorazepam, Lortab, Lotrim, Lovenox, Maalox, Magnesium, Mantoux, Melatonin, Metformin, Methadone, Metoclopramide, Metoprolol, Montelukast, Morphine, Motrin, Mupirocin, Mycostatin, Nafallin, Namenda, Naphazoline, Naphcon, Narco, Neomycin, Neurontin, Nexium, Norvasc, Novolin, Novolog, Nuvigil, Nystatin, Omeprazole, Ondansethron, Pantaprazole, Pantoprazole, Peridex, Phenadoz, Phenergan, Piperacillin, Pipevacillin, Pneumococal polysaccharide vaccine, Polyethylene, Polyethylene Glycol, Polyvinil, Potassium Chloride, Potonix, Prednisone, Prevacid, Prilosec, Primaxin, Promethazine, Propranolol, Protonix, Proventil, Provigil, Qentamicin, Ramipril, Ranitidine, Reglan, Regular insulin, Relistor, Resveratrol, Risperdal, Ritalin, Robinul, Robitussin, Rocephin, Scopolamine, Selenium, Semithicone, Senexon, Senna, Senna-lax, Sertraline, Silace, Siltussin, Simethicone, Simvastatin, Sinemet, Singulair, Sitagliptin, Sodium Chloride, Soma, Sucralfate, Sulfamethoxazole, Sulfreetamide, Symmetrel, Symmetril, Systane, Tamsulosin, Tenormin, Ternormin, Theophylline, Theraplus liquid, Tizanidine, Tobramycin, Toradol, Tramadol, Transderm Scopolamine, Tretinoin, Triamcinolone, Tubersol, Tussin, Tylenol, Ultram, Valium, Valsartan, Vancomycin, Venlafaxine, Vicodin, Vimpat, Vitamin C, Vitamin D, Warfarin, Xeroform, Xopenex, Zafirlukast, Zanaflex, Zinc Oxide, Zinc sulfate, Zofran	Carbamazepine, Clobazam, Clonazepam, Lacosamide, Lamotrigine, Levetiracetam, Oxcarbazepine, Phenobarbital, Phenytoin, Topiramate, Valproic acid

Supplementary Table 3. Estimates for the efficacy of seven drugs in improving outcome from coma were extracted from a range of controlled trials and case reports. We only included drugs for which evidence suggests some efficacy in recovery following both traumatic and non-traumatic brain injury in the intensive care unit. Note that anatibant and progesterone do not have human efficacy data for non-TBI, but both have shown efficacy in animal stroke models[26,27], and so are included here. Efficacy scores were calculated using the same method as in Table 2, and, where possible, were based on the difference between improvements in the placebo versus drug groups. Note that a negative raw score change in the Cerebral Performance Category indicates improvement of a patient's condition, and that this is reflected as a positive value in the normalized score change. GCS=Glasgow Coma Scale, CPC=Cerebral Performance Category, GOS=Glasgow Outcome Scale, Mathew=Mathew stroke scale, NIHSS=NIH Stroke Scale/Score.

**Drug**	**Ref**	**Outcome measure**	**Placebo-controlled**	**Etiology**	**Raw score change**	**Normalized change**
Amantadine	28	GCS	yes	Ischemic stroke, intracerebral haemorrhage, subarachnoid haemorrhage, bacterial meningitis, status epilepticus	3	0.25
	29	CPC	yes	Coma post cardiac arrest	-0.229	0.0573
	30	GCS	no	TBI	2.5	0.208
						**Average= 0.172**
Modafinil	31	% Responders	no	Ischemic stroke, intracerebral haemorrhage, subarachnoid haemorrhage		0
	32	Discharge Disposition	yes	Stroke	13.8	0.138
	30	GCS	no	TBI	3	0.25
						**Average= 0.129**
Nimodipine	33	GOS	yes	TBI	1.6	0.4
	34	GCS	yes	Subarachnoid hemorrhage	1.24	0.103
	35	Mathew	yes	Ischemic stroke	11.6	0.116
						**Average= 0.206**
Methylphenidate	29	CPC	yes	Coma post cardiac arrest	-0.563	0.141
	30	GCS	no	TBI	1	0.083
						**Average= 0.112**
Memantine	36	NIHSS	yes	Ischemic stroke	1.72	0.041
	37	GCS	yes	Moderate TBI	1.4	0.117
	38	GCS	yes	TBI	1.02	0.085
						**Average= 0.081**
Anatibant	39	GOS	yes	TBI	0.75	0.188
						**Average= 0.188**
Progesterone	40	GOS	yes	TBI	0.615	0.154
						**Average= 0.154**

Supplementary Table 4. Drugs and supplements that are ineffective, or presumed to be ineffective, in restoring awareness in acute DOC. Data are based on previously published reports (right column), or on a retrospective clinical analysis using the Discovery Data Repository at UCLA Health (left column). For the retrospective analysis, we specifically looked for medications that were taken by acute DOC patients (Glasgow Coma Scale less than 9 in the intensive care unit) who failed to recover consciousness, and which were not taken by any acute DOC patients who successfully recovered consciousness.

**Drugs from Patient Records**	**Drugs from Previously Published Trials**
Adenosine, Baclofen, Benzocaine, Caffeine citrate, Ceftaroline fosamil, Cisatracurium, Clonazepam, Diltiazem, Docusate sodium, Doravirine, Epinephrine, Eplerenone, Esomeprazole, Estradiol, Ethinyl estradiol, Fentanyl, Gentamicin, Glycerin, Heparin, Hydrochlorothiazide, Hydrocortisone, Ioversol, Ixazomib, Ketamine, Lamotrigine, Leuprolide, Levocarnitine, Levonorgestrel, Levothyroxine, Lurasidone, Lysine, Mitomycin, Morphine, Nitroglycerin, Nizatidine, Pentobarbital, Phenytoin, Propylthiouracil, Pyrazinamide, Risperidone, Sodium phenylbutyrate, Somatropin, Treprostinil, Triamterene, Trimethoprim, Vasopressin	Dexamethasone[41], Dexanabinol[42], Magnesium sulphate[43,44], Mannitol[45], Methylprednisolone[46], Nicardipine[47], Rosuvastatin[48], Selfotel[49], Tirilazad[50]

References

[1] Zafonte, R., Watanabe, T. & R. Mann, N. Case study: Amantadine: a potential treatment for the minimally conscious state. *Brain injury* **12**, 617–621 (1998).

[2] Schnakers, C. *et al.* Measuring the effect of amantadine in chronic anoxic minimally conscious state. *Journal of Neurology, Neurosurgery & Psychiatry* **79**, 225–227 (2008).

[3] Giacino, J. T. *et al.* Placebo-controlled trial of amantadine for severe traumatic brain injury. *New England Journal of Medicine* **366**, 819–826 (2012).

[4] Clauss, R. & Nel, W. Drug induced arousal from the permanent vegetative state. *NeuroRehabilitation* **21**, 23–28 (2006).

[5] Shames, J. L. & Ring, H. Transient reversal of anoxic brain injury- related minimally conscious state after zolpidem administration: a case report. *Archives of physical medicine and rehabilitation* **89**, 386–388 (2008).

[6] Whyte, J. & Myers, R. Incidence of clinically significant responses to zolpidem among patients with disorders of consciousness: a preliminary placebo controlled trial. *American journal of physical medicine & rehabilitation* **88**, 410–418 (2009).

[7] Snyman, N. *et al.* Zolpidem for persistent vegetative state–a placebo-controlled trial in pediatrics. *Neuropediatrics* **41**, 223–227 (2010).

[8] Whyte, J. *et al.* Zolpidem and restoration of consciousness. *American journal of physical medicine & rehabilitation* **93**, 101–113 (2014).

[9] Thonnard, M. *et al.* Effect of zolpidem in chronic disorders of consciousness: a prospective open-label study. *Functional Neurology* **28**, 259 (2013).

[10] Fridman, E. A. *et al.* Fast awakening from minimally conscious state with apomorphine. *Brain injury* **23**, 172–177 (2009).

[11] Fridman, E. A. *et al.* Continuous subcutaneous apomorphine for severe disorders of consciousness after traumatic brain injury. *Brain injury* **24**, 636–641 (2010).

[12] Sanz, L. R. *et al.* Apomorphine for prolonged disorders of consciousness: a multimodal open-label study. *eClinicalMedicine* **78** (2024).

[13] Passler, M. A. & Riggs, R. V. Positive outcomes in traumatic brain injury–vegetative state: Patients treated with bromocriptine. *Archives of physical medicine and rehabilitation* **82**, 311–315 (2001).

[14] Morgado, J., Branco, P., Castanho, P. & Manita, M. Awakening after initiating levodopa in a thalamic hemorrhage. *Journal of the Neurological Sciences* **333**, e283 (2013).

[15] Ugoya, S. O. & Akinyemi, R. O. The place of levodopa/carbidopa in persistent vegetative state. *Psychophysiology* (2010).

[16] Sarà, M., Pistoia, F., Mura, E., Onorati, P. & Govoni, S. Intrathecal baclofen in patients with persistent vegetative state: 2 hypotheses. *Archives of physical medicine and rehabilitation* **90**, 1245–1249 (2009).

[17] Margetis, K. *et al.* Intrathecal baclofen associated with improvement of consciousness disorders in spasticity patients. *Neuromodulation: Technology at the Neural Interface* **17**, 699–704 (2014).

[18] Formisano, R. *et al.* Late recovery of responsiveness after intra-thecal baclofen pump implantation and the role of diffuse pain and severe spasticity: a case report. *Acta Neurochirurgica* **161**, 1965–1967 (2019).

[19] Halbmayer, L.-M. *et al.* On the recovery of disorders of consciousness under intrathecal baclofen administration for severe spasticity—An observational study. *Brain and Behavior* **12**, e2566 (2022).

[20] Reinhard, D. L., Whyte, J. & Sandel, E. Improved arousal and initiation following tricyclic antidepressant use in severe brain injury. *Archives of physical medicine and rehabilitation* **77**, 80–83 (1996).

[21] Worzniak, M., Fetters, M. D. & Comfort, M. Methylphenidate in the treatment of coma. *Journal of family practice* **44**, 495–498 (1997).

[22] Kim, Y. W., Shin, J.-C. & An, Y.-s. Effects of methylphenidate on cerebral glucose metabolism in patients with impaired consciousness after acquired brain injury. *Clinical neuropharmacology* **32**, 335–339 (2009).

[23] Dhamapurkar, S. K., Wilson, B. A., Rose, A., Watson, P. & Shiel, A. Does Modafinil improve the level of consciousness for people with a prolonged disorder of consciousness? A retrospective pilot study. *Disability and Rehabilitation* **39**, 2633–2639 (2017).

[24] Masotta, O., Trojano, L., Loreto, V., Moretta, P. & Estraneo, A. Selegiline in patients with disorder of consciousness: An open pilot study. *Canadian Journal of Neurological Sciences* **45**, 688–691 (2018).

[25] Bagnato, S., Boccagni, C., Sant'Angelo, A. & Galardi, G. A range of antiepileptic drugs do not affect the recovery of consciousness in vegetative and minimally conscious states. *Epilepsy & Behavior* **27**, 365–370 (2013).

[26] Albert-Weißenberger, C., Sirén, A.-L. & Kleinschnitz, C. Ischemic stroke and traumatic brain injury: The role of the kallikrein–kinin system. *Progress in neurobiology* **101**, 65–82 (2013).

[27] Wali, B., Ishrat, T., Won, S., Stein, D. G. & Sayeed, I. Progesterone in experimental permanent stroke: a dose-response and therapeutic time-window study. *Brain* **137**, 486–502 (2014).

[28] Rühl, L. *et al.* Amantadine treatment is associated with improved consciousness in patients with non-traumatic brain injury. *Journal of Neurology, Neurosurgery & Psychiatry* **93**, 582–587 (2022).

[29] Reynolds, J. C., Rittenberger, J. C. & Callaway, C. W. Methylphenidate and amantadine to stimulate reawakening in comatose patients resuscitated from cardiac arrest. *Resuscitation* **84**, 818–824 (2013).

[30] Oommen, J. K. *et al.* Efficacy of modafinil, methylphenidate, amantadine, and zolpidem in consciousness recovery in intensive care unit patients with traumatic brain injury. *PT* **44**, 676–81 (2019).

[31] Leclerc, A. M. *et al.* Amantadine and modafinil as neurostimulants following acute stroke: a retrospective study of intensive care unit patients. *Neurocritical Care* **34**, 102–111 (2021).

[32] Cross, D. B. *et al.* Modafinil in recovery after stroke (MIRAS): a retrospective study. *Journal of Stroke and Cerebrovascular Diseases* **29**, 104645 (2020).

[33] Aslan, A., Gurelik, M., Cemek, M., Goksel, H. M. & Buyukokuroglu, M. E. Nimodipine can improve cerebral metabolism and outcome in patients with severe head trauma. *Pharmacological research* **59**, 120–124 (2009).

[34] Liu, J. *et al.* Efficacy of nimodipine in the treatment of subarachnoid hemorrhage: a meta-analysis. *Arquivos de Neuro-Psiquiatria* **80**, 663–670 (2022).

[35] Gelmers, H. J., Gorter, K., de Weerdt, C. J. & Wiezer, H. J. A controlled trial of nimodipine in acute ischemic stroke. *New England Journal of Medicine* **318**, 203–207 (1988).

[36] Moghadam, N. B. *et al.* The effects of memantine on the serum concentrations of matrix metalloproteinases and neurologic function of patients with ischemic stroke. *Journal of Clinical Neuroscience* **90**, 268–272 (2021).

[37] Mokhtari, M. *et al.* Effect of memantine on serum levels of neuron-specific enolase and on the Glasgow Coma Scale in patients with moderate traumatic brain injury. *The Journal of Clinical Pharmacology* **58**, 42–47 (2018).

[38] Ramezani, A. *et al.* The Effects of Memantine on the Glasgow Coma Scale, Sequential Organ Failure Assessment Score, and Neuron-Specific Enolase Serum Levels in Traumatic Brain Injury Patients. *Journal of Nursing and Midwifery Sciences* **10** (2023).

[39] Marmarou, A. *et al.* A single dose, three-arm, placebo-controlled, phase I study of the bradykinin B2 receptor antagonist Anatibant (LF16-0687Ms) in patients with severe traumatic brain injury. *Journal of Neurotrauma* **22**, 1444–1455 (2005).

[40] Xiao, G., Wei, J., Yan, W., Wang, W. & Lu, Z. Improved outcomes from the administration of progesterone for patients with acute severe traumatic brain injury: a randomized controlled trial. *Critical care* **12**, 1–10 (2008).

[41] Kaktis, J. & Pitts, L. Complications associated with the use of megadose corticosteroids in head-injured adults. *Journal of Neurosurgery Nursing* **12**, 166–171 (1980).

[42] Maas, A., Murray, G., Henney, H. *et al.* Efficacy and safety of dexanabinol in severe traumatic brain injury: results of a phase III randomised, placebo-controlled, clinical trial. *Lancet Neurology* **5**, 38–45 (2006).

[43] Temkin, N., Anderson, G., Winn, H. *et al.* Magnesium sulfate for neuroprotection after traumatic brain injury: a randomised controlled trial. *Lancet Neurology* **6**, 29–38 (2007).

[44] Natale, J. E. *et al.* Pilot study to determine the hemodynamic safety and feasibility of magnesium sulfate infusion in children with severe traumatic brain injury. *Pediatric Critical Care Medicine* **8**, 81 (2007).

[45] Vialet, R. *et al.* Isovolume hypertonic solutes (sodium chloride or mannitol) in the treatment of refractory posttraumatic intracranial hypertension: 2 mL/kg 7.5% saline is more effective than 2mL/kg 20% mannitol. *Critical Care Medicine* **31**, 1683–1687 (2003).

[46] Giannotta, S., Weiss, M., Apuzzo, M. & Martin, E. High dose glucocorticoids in the management of severe head injury. *Neurosurgery* **15**, 497–501 (1984).

[47] Langham, J., Goldfrad, C., Teasdale, G. *et al.* Calcium channel blockers for acute traumatic brain injury. *Cochrane Database of Systematic Reviews* (2003).

[48] Tapia-Perez, J., Sanchez-Aguilar, M., Torres-Corzo, J. *et al.* Effect of rosuvastatin on amnesia and disorientation after traumatic brain injury (NCT003229758). *Journal of Neurotrauma* **25**, 1011–1017 (2008).

[49] Morris, G., Bullock, R., Marshall, S. *et al.* Failure of the competitive N-methyl-D-aspartate antagonist Selfotel (CGS 19755) in the treatment of severe head injury: results of two phase III clinical trials. *Journal of Neurosurgery* **91**, 737–743 (1999).

[50] Marshall, L., Maas, A., Marshall, S. *et al.* A multicenter trial on the efficacy of using tirilazad mesylate in cases of head injury. *Journal of Neurosurgery* **89**, 519–525 (1998).
